# Homeostatic role of B-1 cells in tissue immunity

**DOI:** 10.3389/fimmu.2023.1106294

**Published:** 2023-09-08

**Authors:** Ondrej Suchanek, Menna R. Clatworthy

**Affiliations:** ^1^ Molecular Immunity Unit, Department of Medicine, University of Cambridge, Cambridge, United Kingdom; ^2^ NIHR Cambridge Biomedical Research Centre, Cambridge University Hospitals NHS Foundation Trust, Cambridge, United Kingdom; ^3^ Wellcome Sanger Institute, Hinxton, United Kingdom

**Keywords:** B-1 cells, innate-like B cells, tissue-residency, homeostasis, non-lymphoid organ, tissue immunity

## Abstract

To date, studies of tissue-resident immunity have mainly focused on innate immune cells and T cells, with limited data on B cells. B-1 B cells are a unique subset of B cells with innate-like properties, enriched in murine pleural and peritoneal cavities and distinct from conventional B-2 cells in their ontogeny, phenotype and function. Here we discuss how B-1 cells represent exemplar tissue-resident immune cells, summarizing the evidence for their long-term persistence & self-renewal within tissues, differential transcriptional programming shaped by organ-specific environmental cues, as well as their tissue-homeostatic functions. Finally, we review the emerging data supporting the presence and homeostatic role of B-1 cells across non-lymphoid organs (NLOs) both in mouse and human.

## 1 Introduction

Our understanding of mammalian immunity is largely based on studying immune cells in blood and lymphoid organs. However, there is a growing appreciation that some subsets of innate and adaptive immune cells reside permanently in non-lymphoid organs (NLOs) without recirculation. To date, studies of these tissue sentinels have mainly focused on innate immune cells and T cells, with limited data on B cells (1–5). Here we discuss B-1 cells, a subset of B cells with “innate-like” properties that have several features ascribed to tissue-resident lymphocytes. We review the current evidence for their presence and homeostatic role in NLOs in mouse and human.

B-1 B cells represent a unique subset of B cells that is distinct from conventional B-2 cells in terms of their ontogeny, phenotype and function (6, 7). Murine B-1 cells were first discovered as Ly-1^+^ (now CD5^+^) B cells in early 1980s by Kyoko Hayakawa in Herzenberg’s lab at Stanford University, fueled by the motivation to find the physiological counterpart to human CD5-expressing chronic B-cell leukemia (B-CLL) cells (8, 9). The “B-1” label denotes the fact that their development begins early in ontogeny - preceding B-2 cells. Although the original search for CD5^+^ B cells led to the identification of this unique subset, later studies reported B cells with B-1 characteristics but lacking CD5 expression. Thus, B-1 cells in mouse, identified as B220^low^IgM^hi^CD23^-^CD43^+^IgD^low^, have been further sub-classified as B-1a (CD5^+^) and B-1b (CD5^-^). While B-1a cells are preferentially produced by yolk-sac and foetal liver precursors, B-1b cells dominate the post-natal generation of B-1 cells in bone marrow (10, 11). Although some studies attempted to explain the existence of two B-1 subsets by the “division of labour” model, for example, the generation of natural versus antigen-driven antibody production in B-1a and B-1b, respectively, others did not observe such a functional split (12–14). Moreover, Savage *et* al. showed that upon toll-like receptor (TLR) stimulation B-1a B cells down-regulated their CD5 expression, suggesting B-1b cells being simply an activated form of B-1a cells rather than a distinct subset (15).

Although most of the evidence to date examining B-1 cells has utilized murine systems, and their existence in humans has been a matter of controversy, some recent studies applying single cell genomics to pre-natal human cells have clarified aspects of cross-species similarity (16). Here, we will initially focus on the body of work from mouse models, and then turn to human studies of innate-like B cells.

## 2 Tissue-residency of B-1 cells

**Figure 1:**
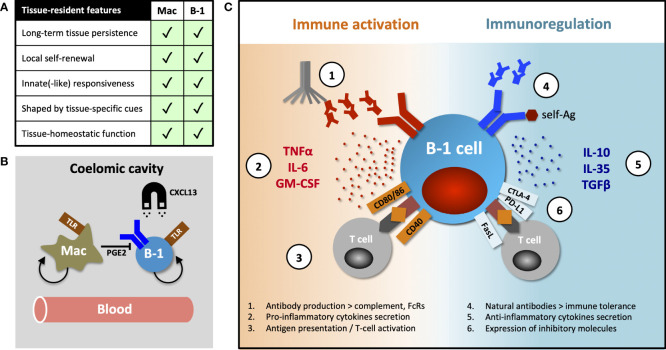
Tissue-resident features of B-1 cells and their diverse functions. **(A)** Table comparing canonical features of tissue residency between macrophages and B-1 cells. **(B)** B-1 cell innate-like responsiveness is mediated by TLR signaling and their tissue homing, at least in part, by CXCL13. Peritoneal macrophages influence nearby B-1 cells *via* prostaglandin E2 (PGE2), inhibiting their natural IgM production. **(C)** B-1 cells perform their diverse roles (including homeostatic functions) *via* both antibody-dependent and antibody–independent (cytokines secretion, antigen-specific activation or inhibition of T cells) mechanisms. They promote or regulate immune responses by other immune cells in direct contact-dependent or –independent (by-stander) mechanisms. Contact-dependent interaction with T cells can be antigen-specific, relying on coupling MHC/TCR coupling. While antibodies modulate immune response through complement activation and binding activating and inhibitory Fc-receptors (FcRs), they (in particular, natural antibodies) also play an essential role in maintaining both central and peripheral immune tolerance.

Tissue-resident immune cells have several canonical features, exemplified and first demonstrated in macrophages, namely long-term persistence & self-renewal within tissues, differential transcriptional programming shaped by organ-specific environmental cues and tissue-homeostatic functions. These characteristics were subsequently described in tissue-resident T- and NK cells (reviewed by Fan and Rudensky (17)), but here we posit that B-1 cells also exhibit several of these features (**Figure 1A**).

Murine B-1 cells predominantly reside in pleural and peritoneal cavities where they constitute 35-70% of local B cells. A smaller proportion of these cells can also be found in the spleen, bone marrow, lymph nodes and some NLOs (1, 6, 18, 19). Ansel et al. demonstrated that almost 70% of peritoneal B-1 cells remained sessile after 8-week parabiosis and that their homing into the peritoneal cavity was mediated, at least in part, by CXCL13 (20) (**Figure 1B**). The fact that ~30% of peritoneal B-1 cells exchanged in their parabiosis experiment, and that B-1 cells are detectable in peripheral blood, indicate that some B-1 cells undergo homeostatic re-circulation (6, 14, 20). However, little is known about their patterns of migration and the molecular mechanisms underpinning their dynamic behavior. Certainly, innate stimulation (e.g. *via* TLR), results in B-1 cell migration from body cavities into secondary lymphoid organs, a phenomenon that seems to be CD11b- and CD6-dependent (14, 21–23).

In addition to high CD11b and low CD6 expression, peritoneal B-1 cells differ from their counterparts in secondary lymphoid organs in terms of their transcriptional profile, immune repertoire (for example, they have a higher frequency of *Ighv11*-encoded phosphatidyl-choline binders) and inability to secrete natural IgM antibodies (24–27). Adoptive transfer studies, together with the evidence for B-1 cell re-circulation, show site-specific differences in phenotype and function and indicate substantial B-1 cell plasticity and an ability to adapt to organ-specific cues, rather than the existence of several distinct B-1 subsets preferentially seeding different tissues (26, 27). Chace *et* al. provided a good example of such a tissue-specific influence, showing that peritoneal macrophages inhibit IgM secretion of nearby B-1 cells *via* prostaglandin E2 secretion (28) (**Figure 1B**).

## 3 Self-renewing capacity and innate-like responsiveness of B-1 cells

An important B-1 cell feature is the capacity to self-renew in situ, as suggested by the ability of adoptively transferred mature peritoneal B-1 cells to reconstitute all B-1 cell compartments (10, 29–32). Recently, Clark *et* al. showed that B-1 cell maintenance and self-renewal in the lipid-rich peritoneal cavity is dependent on autophagy and a specific metabolic programme characterized by fatty acid synthesis, oxidative phosphorylation, but also high levels of glycolysis (33). B-1 cells were able to utilize and store exogenous lipids available in the surrounding microenvironment, further demonstrating their metabolic adaptation to their tissue of residence (33). B-1 cells have unique intracellular signaling characteristics that distinguish them from B-2 cells and are essential for their long-term survival. These include a relative unresponsiveness to BCR crosslinking (evidenced by an inability to mobilize intracellular calcium and a lack of NF-κB activation and proliferation) and active basal tonic signaling downstream of the BCR with constitutive phosphorylation of SYK, ERK and STAT3, which occurs in B-2 cells only after BCR stimulation (34–36). This relative inhibition of BCR-induced proliferation is controlled by the balanced expression of positive (for example, CD19) and negative (for example, CD5, Siglec-G) BCR regulators, the perturbation of which also affected B-1 cell survival (32, 37–39). However, the ligands for these BCR regulators still remain to be found and/or confirmed. Anergic B-2 cells may share some of these characteristics (e.g. unresponsiveness of low-level self-reactive BCRs, constitutive ERK phosphorylation or CD5 expression) (40). However, in contrast to anergic B-2 cells, B-1 cells live longer (both *in vitro* and *in vivo*), express higher levels of surface IgM and co-stimulatory molecules, and upon BCR cross-linking phosphorylate AKT and upregulate MHCII (35, 40–42). Interestingly, unlike B-2 cells, B-1 cell development and survival does not seem to be dependent on the myeloid-derived cytokine BAFF (B cell activating factor of the TNF receptor family). Although Lam et al. demonstrated its enhancing role in B-1 cell activation, leading to increased expression of CD21/35 and NF-κB activation (43, 44).

One hallmark of tissue-resident lymphocytes is their innate-like ability to recognize danger and memory/memory-like phenotype, enabling rapid responses. B-1 cells vigorously respond to innate stimuli, for example mediated *via* TLR or interleukin (IL)5 receptor engagement, leading to their emigration from body cavities into secondary lymphoid organs and to rapid up-regulation of BLIMP1 and their subsequent differentiation into plasma cells, as also observed in marginal zone (MZ) B cells (21, 45, 46). The strong B-1 cell response to innate stimulation and relative BCR unresponsiveness might imply that B-1 cell activation is independent of BCR signaling. However, Savage et al. demonstrated that TLR stimulation in B-1 cells led to the reorganization of their BCR signalosome (including association with CD19 and loss of CD5) which, as a licensing step, released the initial BCR signaling block present in resting B-1 cells (15).

## 4 Homeostatic function of B-1 cells

One of the key functions of B-1 cells is their homeostatic “spontaneous” production of natural antibodies (predominantly IgM and IgG3), even in the absence of any foreign microbial stimuli (47). Recently, Zeng *et* al. reported the role of sex hormones in natural antibody production. They found that the female advantage in the clearance of enteropathogenic Escherichia coli was driven by oestrogen-dependent production of natural antibodies that bind the bacterium (48). IL-5, an inducer of BLIMP1 expression, is another factor shown to play a role in tonic antibody secretion (46). As noted above, natural antibodies are produced outside body cavities, mostly in the bone marrow and spleen (26). Savage *et* al. demonstrated that the population of natural antibody-secreting cells is heterogeneous, containing a significant proportion of non-terminally differentiated, BLIMP1^neg^ cells (49). Recently, Benezech *et* al. added further complexity to this model of compartmentalized natural IgM secretion, exploring antibody production in fat-associated lymphoid clusters (FALCs) and milky spots located in serous cavities (50). These small anatomical niches are a source of CXCL13, IL33 (from stromal cells) and IL5 (from group 2 innate-lymphoid cells (ILC2), and sites of pleural and peritoneal B-1 cell activation, both during homeostasis and infection; allowing local production of IgM for protection of these body cavities (51, 52).

Natural antibodies are polyreactive, able to react with both self- (PtC, Thy1) and microbial (e.g. pneumococcus or influenza) antigens (13, 53, 54). They provide an instant defense against invading pathogens, for example, by direct neutralization or complement binding, but also promote B-2 antigen-specific responses, possibly by forming immune complexes that are more easily trapped by antigen presenting cells or follicular dendritic cells (13, 55). Natural antibodies also participate in tissue homeostasis by opsonizing and enhancing the clearance of apoptotic cells or low-density lipoproteins by mononuclear phagocytes (56, 57). There is a good body of evidence that B-1 cells also substantially contribute to the production of mucosal IgA, maintaining the symbiotic homeostatic relationship between the host and microbiome, as reviewed by Almut Meyer-Bahlburg (58). However, whether these antibodies are still “natural”, i.e. produced independently of microbial antigens, is hotly debated (58). In addition to the steady-state secretion of natural antibodies, B-1 cells can actively respond to a variety of pathogens or tumor antigens with increased (induced) IgM production following activation (59, 60). There is also some evidence that IgM natural antibodies enforce central tolerance and promote normal development of both B-1 and B-2 cells by a mechanism that is not completely clear, possibly by facilitating the tolerogenic presentation of autoantigens (61). On the other hand, natural antibodies can be an integral part of immunopathological processes such as ischaemia-reperfusion injury or the delayed hypersensitivity response in skin (62, 63).

B-1 cells have been shown to regulate immune response by mechanisms other than antibody secretion, including cytokine production. At steady state, a subset of peritoneal, and to a lesser extent splenic, B-1 cells spontaneously secretes the regulatory cytokine IL10 (64). A protective role for B-1 cell-derived IL10 has been demonstrated in an endotoxaemic-induced systemic inflammatory response and in autoimmune-prone mice (65–67). In addition to IL10, B-1 cells can also exert their regulatory function by secretion of IL35 and transforming growth factor beta (TGFβ) (68, 69) or in contact-dependent manner by expression of inhibitory molecules FasL, PD-L2 and CTLA-4 (in particular, B-1 cells with autoreactive specificities) (70–73).

Conversely, Rauch and Weber *et* al. described a B-1 cell subset they termed “innate response activator” (IRA) that upon TLR stimulation, migrated from body cavities to spleen or lung to enhance acute inflammatory responses *via* granulocyte macrophage colony stimulating factor (GM-CSF) and IL3 secretion, in addition to IgM (74, 75). Others observed that peritoneal B-1 cells stimulated with LPS increased both IL10 and IL6 secretion, proposing that the ratio between these cytokines ultimately determines whether their effect on co-cultured CD4 T cells is activating or inhibitory (76). Finally, B-1 cells may also act as efficient antigen-presenting cells and activators of T cells through their high constitutive expression of MHCII and CD80/86, as reviewed by Popi et al. (77).

Together these data indicate that B-1 cells are a functionally heterogeneous population capable of both immune suppression and stimulation (**Figure 1C**). It is unclear how much functional plasticity exist in any given B-1 cell for these two opposing roles and whether under certain circumstances all B-1 cells can eventually mount a pro-inflammatory response to infection.

## 5 Presence of B-1 cells in NLOs

**Figure 2:**
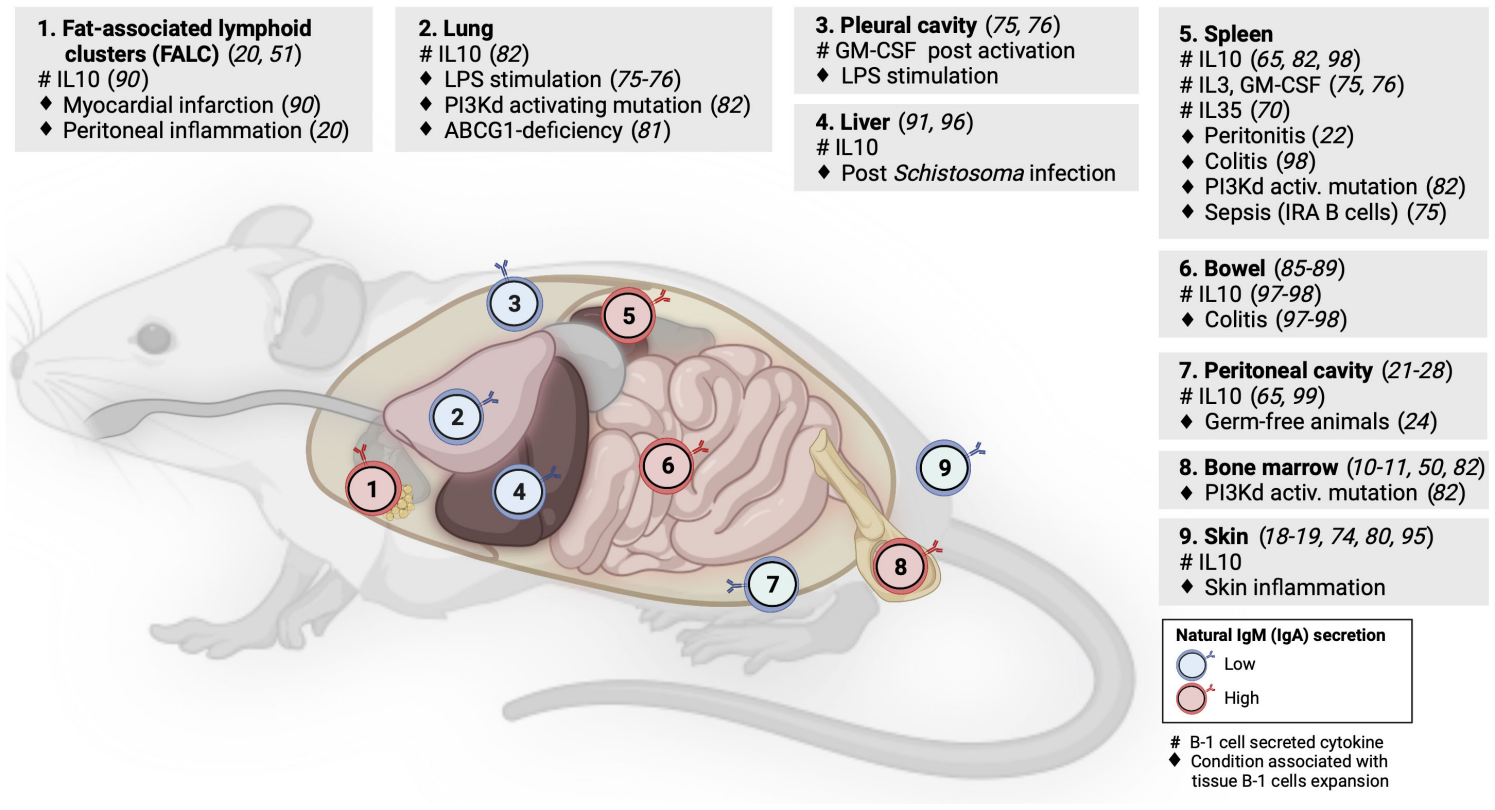
B-1 cell distribution across mouse lymphoid and non-lymphoid organs. Numbers within B-cell icons label mouse organs where B-1 cells have been documented. B-cell icon color indicates whether intra-organ production of natural antibodies is low (blue) or high (red). Grey panels show cytokines produced by tissue B-1 cells (#) and conditions associated with expansion of tissue B-1 cells (♦), linked literature references numbers are in brackets.

Although some studies of tissue-resident memory B cells or natural antibody-producing plasma cells have identified B-1 B cells in NLOs at steady state (5, 78), these NLO-resident B-1 cells have received limited attention to date. However, evidence suggests the homeostatic presence of B-1 cells in skin, lungs, gut, liver and visceral fat, in addition to pleural and peritoneal cavities (**Figure 2**).

Geherin et al. used afferent lymphatic cannulation in sheep to demonstrate the presence of innate-like (IgM^hi^CD11b^hi^) B cells trafficking from uninflamed skin (18). Subsequently, they reported IL10^+^ B-1 B cells residing both in murine and human skin under normal conditions (1). Baldan et al. observed an expansion of B-1 cells in the lungs of ATP-binding cassette transporter G1 (ABCG1)-deficient mice as a result of lipid accumulation and chronic inflammation. These B-1 cells produced natural lipoprotein-binding antibodies that may protect against atherosclerosis (79). Stark et al. found a marked accumulation of aberrant B-1 cells in the lungs of mice with either global or B-cell specific activating mutation in the catalytic subunit of phosphoinositide-3-kinase δ (PI3Kd) that was demonstrated by others to increase the generation of B-1 cells in the bone marrow (80, 81). These lung B-1 cells secreted IL10 and were associated with higher susceptibility to bacterial pneumonia as seen in patients with activated PI3Kδ syndrome (80, 82). Using long-term B cell chimeras, Krosse et al. reported that almost half of IgA secreting plasma cells in the gut were derived from peritoneal B-1 B cells (83). However, this finding was subsequently disputed in gnotobiotic Ig allochimeric mice experiments (84) or by examining *Igavh* repertoire in lamina propria from L2 mice that lack B-2 cells (85). Recently, two studies re-examined this controversy: Bunker et al. performed a series of B-cell transfer experiments into *Rag1* ^-/-^ mice and found that commensal-specific IgA^+^ gut plasma cells originated from B-2 and, to a lesser extent, also from B-1b (but not B-1a) cells (86). Vergani et al. demonstrated that B-1a cells and IgA^+^ gut plasma cells shared the same haematopoietic progenitors in early life using two different lineage-tracing mouse models (87). However, there was no substantial clonal overlap between these two populations, suggesting an early bifurcation likely at the point of antigen selection (87). B-1 cells have been also identified in mouse visceral fat in steady state, forming special lymphoid clusters (19, 88). Zhang *et* al. reported IL10-producing B-1a cells in wild-type mouse liver but their quantification did not account for B cells from blood contamination (89). The presence of B-1 B cells in mouse liver and lung was indirectly supported also by experiments showing that a small number of cells from homogenized organs spontaneously secreted IgM on ELISPOT (49).

## 6 Evidence for homeostatic role of tissue-resident B-1 B cells in NLOs

As discussed above, B-1 cells secreting IL10 and natural antibodies have been identified across several NLOs in steady state and with increased abundance during inflammation, suggesting their anti-inflammatory homeostatic role protecting tissue integrity (**Figure 2**). Skin is an exemplar NLO to demonstrate such a B-1 cell function.

Using radioactive labeling, Geherin et al. showed that B-1 cells migrate from their natural reservoirs in the serous cavities into both non-inflamed, and upon innate stimulation, inflamed skin and intestine, in an α4β1 integrin-dependent manner to provide immunosurveillance and to suppress inflammation (1). The regulatory function of cutaneous B-1 cells is also consistent with several case reports of psoriasis exacerbation after therapeutic B-cell depletion with rituximab or α4-integrin blockade with natalizumab (90–92). Similarly, CD5^+^ IL10^+^ B cells were found to suppress contact hypersensitivity dermatitis in a mouse model (73, 93). A recent study by Wu et al. demonstrated the presence of IL10-producing CD5^+^ B-1a cells in pericardial fat in homeostasis that were subsequently shown to be capable of ameliorating damage associated with myocardial infarction (88). The ameliorating effect of IL10^+^ B-1 cells on local tissue inflammation was also found in the liver after Schistosoma infection (94) and in the gut during acute and chronic colitis (95, 96).

Although the body of evidence for the homeostatic role of IL10 produced by B-1 cells in NLOs organ immunity is increasing, its precise impact on other immune and non-immune cell subsets within tissues is an outstanding question that requires further investigation. In peritoneal cavity, Wong et al. identified that macrophages are polarized toward anti-inflammatory (M2) phenotype by the presence of B-1 cells both *in vitro* and *in vivo* and that this is driven by IL10 but not by IgM (97). They subsequently showed that such B-1 cell driven anti-inflammatory macrophage polarization was detrimental for a skin tumor clearance (97). The regulatory role of tissue innate-like B cells in homeostasis can increase organ susceptibility to infection or be even actively hijacked by pathogens (80, 82). Liu *et* al. reported that Listeria infection stimulates IL10 production by MZ B cells, which in turn, inhibits inducible nitric oxide synthase (iNOS) production in splenic macrophages, leading to an increased bacterial burden (98). In contrast to the anti-inflammatory function of B-1 cells in NLOs at steady state, there is also data for a pro-inflammatory/protective role of B-1 cells in local infection as exemplified by IRA B-1 cells relocating from the pleural cavity into the lung to protect against pneumonia *via* GM-CSF and IgM secretion (75).

As outlined above, most studies examining the role of natural antibodies in tissue homeostasis and protection were based on sampling blood or serous cavities. Savage *et* al. showed that the vast majority of natural IgM-secreting B-1 cells and plasma cells are located in bone marrow and spleen with only less than 5% found in serous cavities and NLOs (49). B-1 cells in peritoneal cavity (outside FALCs/milky spots) are largely suppressed in their natural antibody production by nearby macrophages (28, 50). Although the data on the homeostatic presence of B-1 cells in NLOs suggest some local secretion of natural antibodies, it is unclear if this secretion is inhibited by similar tissue-specific cues as seen in peritoneal cavity.

## 7 Evidence for B-1 cells in human NLOs

The identification of a human equivalent to murine B-1 cells has been a focus of ongoing study, and a source of heated debate and controversy in the field. Early attempts to find human B-1 cells were based on searching for CD5^+^ B cells. As noted previously, the vast majority of human B-CLL cells are CD5 positive. A more detailed analysis of these pathological CD5^+^ B cell clones revealed marked repertoire skewing for auto- and polyreactive BCRs, a feature of murine B-1 cells (99). Recently, Hayakawa *et* al. found a mouse B-1 cell clone with a non-mutated CDR3 region encoding a binding site for non-muscle myosin IIA, which is often a target of human B-CLL BCRs. These B-1 cells expanded with age and eventually became malignant, likely because of the chronic stimulation with a potent auto-antigen (100). Moreover, expanded polyreactive IgM^+^/IgA^+^CD5^+^ B cells were documented in human autoimmune conditions such as rheumatoid arthritis or IgA nephropathy (101–103).

Given the preferential residence of murine B-1 cells in coelomic cavities, human peritoneal washings were also examined for the presence of CD5^+^ B cells. Although the relative proportion of these cells was higher than in blood, their absolute number was negligible compared to mouse (104, 105). Early studies reported a significantly increased frequency of CD5^+^ B cells in human umbilical cord blood, which clearly mirrored findings in mice, in particular with regards to the recently demonstrated role of *Lin28b/Let7* as a switch between fetal and adult hematopoiesis, both in mouse and human (101, 102, 106). However, Lee et al. showed subsequently that the majority of CD5^+^ B cells in human blood represent “pre-naïve” B cells, having an intermediate phenotype between transitional and naïve B cells. Most of these cells lost their CD5 expression upon further differentiation into naïve cells (107). The fact that the frequency of CD5^+^ B cells in human adult blood is almost 20-times higher than in mice and that CD5 is also expressed by transitional, activated or anergic B-2 cells, further support the notion that CD5 is unlikely to be a useful marker for human B-1 cells (108–110).

One effort to define surface markers of putative human B-1 cells was presented by Griffin et al. (111). When profiling human umbilical cord blood they found an unexpectedly high percentage of CD27^+^ “memory” B cells (3-11% of CD20^+^ subset) that also expressed CD43, a canonical marker of murine B-1 cells. Subsequent functional examination of this FACS-sorted subset (CD20^+^CD27^+^CD43^+^) from both neonatal and adult blood confirmed three key B-1 cell characteristics – spontaneous IgM secretion, tonic signaling, and efficient stimulation of T cells (111). The proportion of these putative B-1 cells within CD20^+^CD27^+^ subset clearly declined with age, and only three quarters of these cells were CD5 positive. Since CD43 can be a marker of B-2 cell activation, the authors confirmed that other typical markers of activation (i.e. CD69, CD70) were not expressed by these cells (111). In a subsequent study, they further split CD20^+^CD27^+^CD43^+^ human “B-1” cells into CD11b^-^ “secretor” cells (i.e. primarily secreting natural antibodies) and CD11b^+^ “orchestrator” cells that were expanded in SLE patients, and spontaneously secreted IL10, but could enhance T-cell proliferation and modulate T-cell activation (112, 113). Other groups have struggled to replicate these findings, suggesting potential issues with T cell contamination and doublets and confusion with B-2 pre-plasmablasts (114–116).

Geherin et al. identified B cells in healthy human skin, where 3.9 – 28.6% of them secreted IL10 after four-hour stimulation. Only 3.5% of total skin B cells expressed markers of putative human B-1 B cells (i.e. CD3^-^CD20^+^CD27^+^CD43^+^) discussed earlier (1). Nihal et al. found that B cells are rare in normal human skin, and hence difficult to phenotype, but using PCR-based Ig heavy chain rearrangements analysis, they showed that these B cells had a clonally-restricted BCR repertoire, potentially indicating antigen-driven clonal expansion. These finding suggested the potential existence of tissue-resident B-1 cells in normal human skin (117).

**Figure 3:**
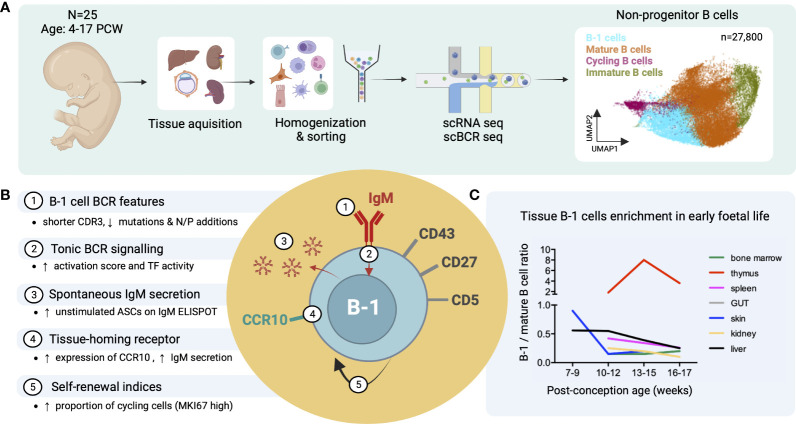
Identification of putative B-1 cells in prenatal human tissues by single cell RNA/BCR sequencing (adapted from Suo et al. (16)). **(A)** Study design schematic: Tissues from 25 human embryos/foetuses aged between 4 and 17 post-conception weeks (PCW) were homogenized into single cell (sc) suspensions, sorted for CD45^+^ live cells and subjected to sc RNA and BCR sequencing (10X Genomics). Total 27,800 non-progenitor B cells were recovered from all processed tissues following quality control (shown in UMAP plot, putative B-1 cells in light blue). **(B)** Characteristics of putative B-1 cells found in this study; mature (IgD^+^IgM^+^CD27^-^) B cells were used as the comparator. CDR3, complementarity-determining region 3; TF, transcription factors; ASC, antibody-secreting cell. **(C)** Plot showing the change of B-1/mature B cell number ratio across different tissues with post-conception age.

Recently, we identified putative B-1 cells in human embryonic and foetal tissues using single cell RNA sequencing (16) (**Figure 3A**). In line with previous reports, these IgM^hi^IgD^low^ B cells were CD27^+^CD43^+^CD5^+^ and shared functional characteristics with murine B-1 cells, including spontaneous antibody secretion and BCR features (reduced CDR3 junction length and NP additions and lower mutation frequency) (**Figure 3B**). Interestingly, by the third trimester, the ratio of B1/mature B cell significantly decreased in most organs except the thymus (**Figure 3C**) (16). The putative B-1 cells expressed also high levels of CCR10 that may play a role in their tissue homing and retention. CCL28, the ligand of CCR10, was expressed in bone marrow stromal cells, and in skin and gut epithelial cells (**Figure 3B**) (16).

Asano et al. reported an accumulation of innate-like B cells in human kidney allografts during rejection that were transcriptionally similar to murine peritoneal B-1 cells, secreted autoantibodies directed against kidney (rather than donor-specific) antigens and expressed a signature gene, *AHNAK* (118). However, it is not clear whether these innate-like B cells expanded locally from cells residing in the kidney in homeostasis or infiltrated from the blood or other reservoir.

Finally, there are several examples that illustrate marked inter-species differences in innate-like tissue-resident lymphocyte populations. For example, iNKT cells constitute the main lymphocyte subset in mouse liver, but they represent less than 1% of human liver lymphocytes (119). γδ T cells are more prominent in bovine skin than mouse or human and significantly differ in their oligoclonality and diversity (120). These studies suggest, that while the functions of innate-like tissue-resident lymphocytes are conserved across species, this common function is not necessarily conserved to specific cell types. Moreover, there is some evidence that innate-like lymphocytes, such as epidermal γδ T cells in mouse skin, might be replaced by Trm after an infection (121). It is plausible that similar phenomena could explain why the equivalent to murine B-1 cells cannot be convincingly identified in adult human tissues (including the peritoneal cavity) as they may have been functionally replaced by other innate-like subsets such as MZ B cells. Indeed, Weller et al. demonstrated marked phenotypic and functional similarity between “IgM memory” B cells in peripheral blood and human MZ B cells suggesting their re-circulation (122). Moreover, human MZ-like B cells were also described in the inner wall of the subcapsular sinus of lymph nodes, tonsillar crypts and mucosa-associated lymphoid tissue (MALT) (123–125).

## 8 Concluding remarks

B-1 cells with their unique combination of innate-like and adaptive functions are well suited to be efficient tissue-resident immune cells. In contrast to B-2 cells, they do not respond to activation by extensive clonal expansion, but rather rapidly migrate, re-distribute and differentiate in a process that seems to be controlled by the receipt of innate immune signals (20, 21, 126). B-1 cells represent an exemplar of how adaptive immune cells residing in NLOs adapt a hybrid functionality with innate and adaptive features, together contributing to the maintenance of local tissue immunity homeostasis and defense. Although tissue-resident innate-like B(-1) cells might have a clear therapeutic potential in a number of intra-organ pathologies (for example, cancer or transplant rejection), future studies should first convincingly delineate their equivalents in human.

## Author contributions

OS wrote the manuscript and designed figures. MRC revised the manuscript and designed figures. All authors contributed to the article and approved the submitted version.
